# Small RNA sequencing reveals distinct nuclear microRNAs in pig granulosa cells during ovarian follicle growth

**DOI:** 10.1186/s13048-021-00802-3

**Published:** 2021-04-20

**Authors:** Derek Toms, Bo Pan, Yinshan Bai, Julang Li

**Affiliations:** 1 Department of Comparative Biology and Experimental Medicine, Faculty of Veterinary Medicine, University of Calgary, Calgary, AB Canada; 2 Department of Animal Biosciences, University of Guelph, Guelph, ON Canada; 3 School of Life Science and Engineering, Foshan University, Foshan, 528231 China

**Keywords:** Microrna, smallRNA, snoRNA, Granulosa cells, Pig, Ovarian, Ovary, Follicle, Reproductive biology, Next generation sequencing, RNA-seq, Cellular fractionation, Nucleus, Cytoplasm

## Abstract

Nuclear small RNAs have emerged as an important subset of non-coding RNA species that are capable of regulating gene expression. A type of small RNA, microRNA (miRNA) have been shown to regulate development of the ovarian follicle via canonical targeting and translational repression. Little has been done to study these molecules at a subcellular level. Using cell fractionation and high throughput sequencing, we surveyed cytoplasmic and nuclear small RNA found in the granulosa cells of the pig ovarian antral preovulatory follicle. Bioinformatics analysis revealed a diverse network of small RNA that differ in their subcellular distribution and implied function. We identified predicted genomic DNA binding sites for nucleus-enriched miRNAs that may potentially be involved in transcriptional regulation. The small nucleolar RNA (snoRNA) SNORA73, known to be involved in steroid synthesis, was also found to be highly enriched in the cytoplasm, suggesting a role for snoRNA species in ovarian function. Taken together, these data provide an important resource to study the small RNAome in ovarian follicles and how they may impact fertility.

**Supplementary Information:**

The online version contains supplementary material available at 10.1186/s13048-021-00802-3.

## Introduction

Increasing ovarian follicle size has long been an important indicator of oocyte quality, with oocytes obtained from large follicles showing consistently higher in vitro and in vivo developmental potential than their counterparts from small follicles [3, 51]. In addition to increased expression of key growth factors like GDF9 and BMP4, we and others have shown differences in microRNA (miRNA) expression in the somatic cells of these follicles [44–46, 54, 55, 57]. Recent investigations have looked at the distinct populations of small RNA that perform key biological functions in the cytoplasmic and nuclear compartments of cells [19, 41, 47, 60]. All of these RNA species are transcribed from chromosomal DNA, and most are processed in both the nucleus and cytoplasm. Accordingly, a complex system of transport exists to dynamically localize small RNA within the cell. For example, after processing into mature miRNA by Dicer in the cytoplasm, strands loaded onto Ago2 may be subsequently imported back into the nucleus via Importin 8 [39]. The availability of complex secondary structures between RNA and DNA species permit these RNA to function in a myriad of ways. In the nucleus, miRNA can form triple helices with chromosomal DNA, to regulate gene expression [48]. Even degraded fragments of miscoded messenger RNA have been shown to participate in the assembly of such scaffolds [9]. Although small nucleolar RNAs (snoRNAs) are principally involved with RNA modification in the nucleus, they are shuttled to the cytoplasm during periods of stress [20, 31]. Despite evidence that such RNA species have functional roles in both the nuclear and cytoplasmic compartments of the cell, little systematic analysis has been done to study them at a population level. Here, we present a survey of small RNA species present in gilt granulosa cells obtained from small and large preovulatory follicles, herein referred to as SGCs and LGCs. While we found a few significant differences between SGCs and LGCs, we did reveal a diverse network of small RNA that showed distinct subcellular localization. This small RNAome of ovarian granulosa cells will provide an important resource for studying their subcellular function during follicle growth.

## Results

### Validation of subcellular fractionation

**Fig. 1:**
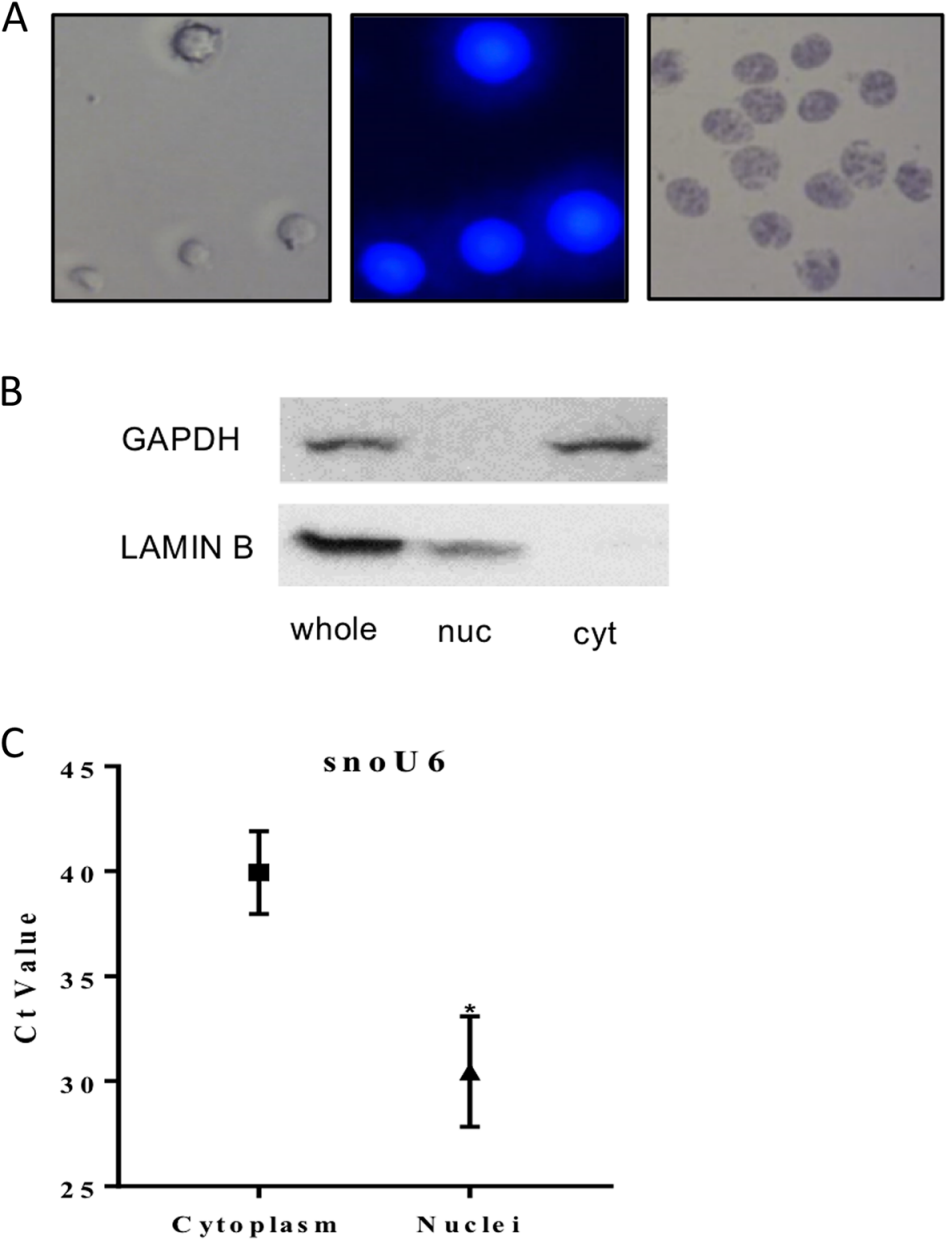
Isolation of nuclear and cytoplasmic fractions. **a** Isolated nuclei were visualized at 400X magnification to confirm purity, left-to-right: phase contrast, Hoescht 33342, and trypan blue. **b** Western blot analysis for whole cell, cytoplasmic (cyt) and nuclear (nuc) extracts from pig granulosa cells using anti-Lamin B and anti-GAPDH antibodies. Lamin B and GAPDH were used as a nuclear or cytoplasmic marker protein, respectively. 50 μg of whole lysate and cytoplasmic extract or 100 μg of nuclear extract was loaded. **c** RT-qPCR of nuclear and cytoplasmic RNA fractions was performed on the small nuclear RNA U6. Data represents the mean ± SEM from three independent experiments

Following cell fractionation, intact nuclei were visualized by phase-contrast and fluorescence microscopy, after chromosome staining with Hoescht 33342 (Fig. 1a). Before proceeding with deep sequencing of small RNAs, we confirmed the quality and purity of the nuclear and cytoplasmic fractions. The RNA integrity number (RIN) for each sample was determined by capillary electrophoresis: LGC cytosol, 8.62 ± 0.40; LGC nucleus, 6.92 ± 0.17; SGC cytosol, 9.02 ± 0.22; SGC nucleus, 6.78 ± 0.46. Western blotting revealed the purity of each fraction by the exclusion of glyceraldehyde 3-phosphate dehydrogenase from the nucleus and lamin B from the cytosol (Fig. 1b). RT-qPCR analysis of the nuclear spliceosomal RNA U6 revealed 1000-fold higher expression in the nucleus, as expected (Fig. 1c), confirming clear separation of the nucleus and cytosol fractions.

### Overview of small RNA sequencing data

**Fig. 2:**
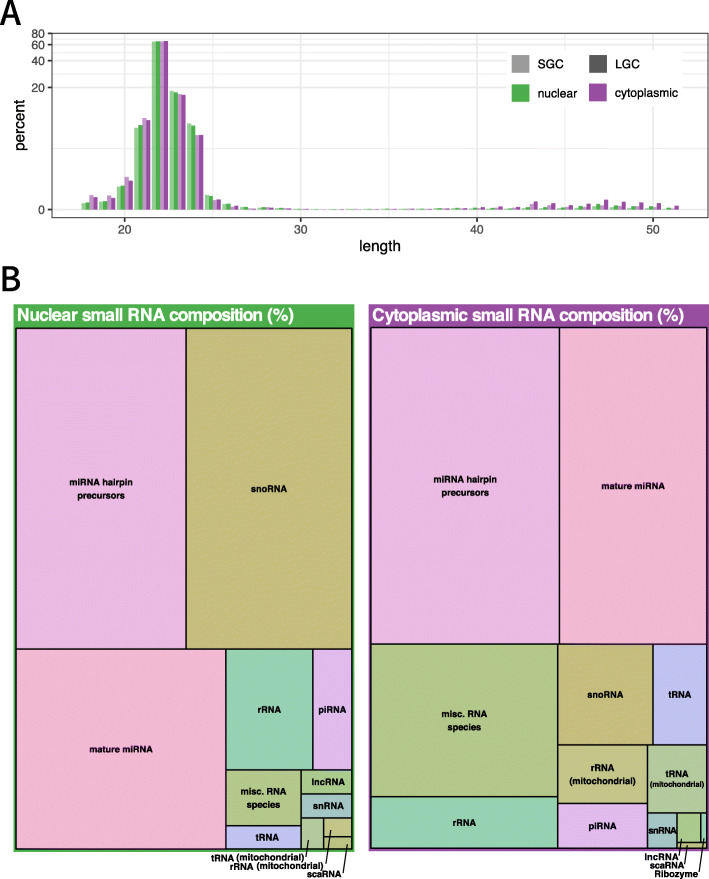
Small RNA read length distribution. **a** Sequenced reads were trimmed to ≥18 nt, and the distribution of the remaining reads for each sample was plotted, showing that 97% of reads had a length of between 21 and 24 nt. **b** Total read counts from pooled cytoplasmic and pooled nuclear fractions (i.e. including both large and small GCs) were aligned to the pig genome and their mapping to various RNA species was graphed. Each rectangle is proportional to the percentage of reads mapped to each category relative to the total read counts

Four nuclear and four cytoplasmic RNA pools were sequenced from both LGCs and SGCs. After trimming adapter sequences, reads with a sequence length less than 18 nt were discarded. Remaining sequencing reads had lengths between 18 and 50 nt (Fig. 2a). The majority of reads (97%) were between 22 and 25 nt in length. Twenty percent of these reads could not be mapped to the pig genome and 7% mapped to coding regions.

We mapped the remaining reads to *S. scrofa* small RNA databases and looked broadly at RNA species breakdown in nuclear and cytoplasmic fractions (Fig. 2b). MicroRNA, both mature sequences and hairpin precursors, made up the majority of the mapped reads in both cell fractions (55% of the nuclear reads; 61% of cytoplasmic reads) followed by 30.4% of nuclear reads mapping to snoRNA, while 16.3% of cytoplasmic reads remained uncharacterized as “miscellaneous RNA”. As expected, considerably fewer cytoplasmic reads mapped to snoRNA, while reads mapping to ribozyme, transfer RNA and mitochondrial RNA were five- to ten-fold lower in the nuclear fraction compared to cytoplasmic fractions.

### MicroRNAs have distinct subcellular localization patterns

**Fig. 3:**
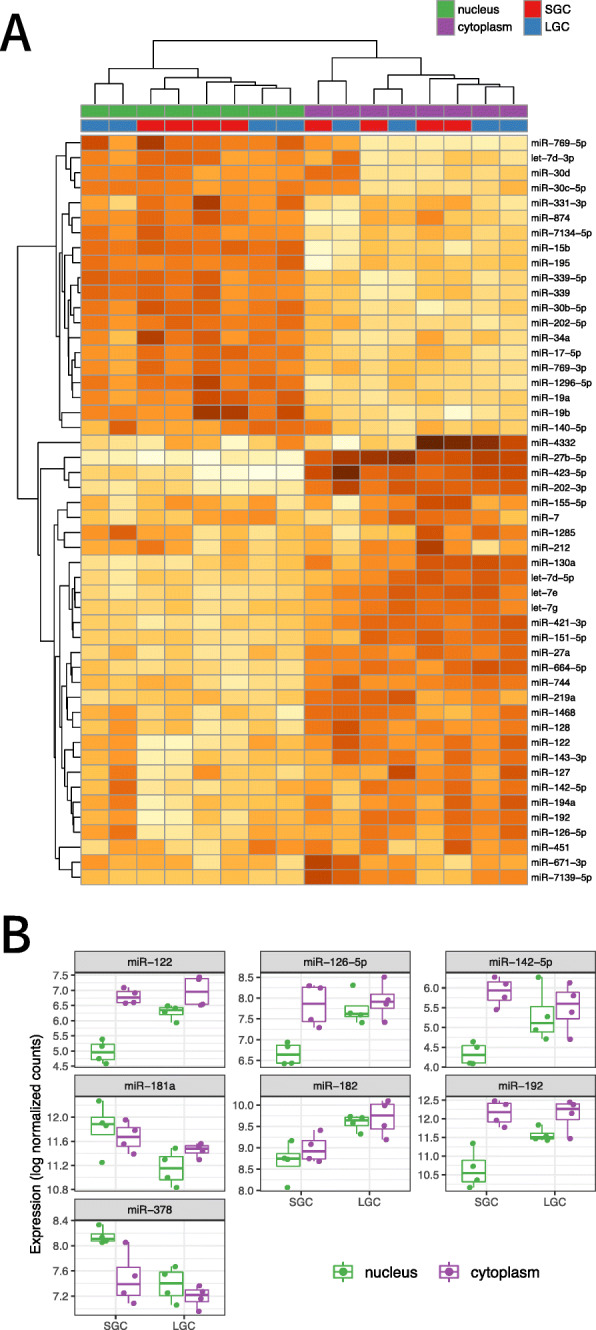
Differential expression of miRNA between the nucleus and cytoplasm in granulosa cells. **a** Unsupervised clustering of the 50 most differentially expressed miRNA between all groups shows distinct expression patterns between the nucleus and the cytoplasm. **b** Box-and-whisker plot of expression values for the miRNAs differentially expressed between SGC and LGC nuclei. Each point represents one biological replicate

After mapping reads to annotated swine miRNA, we looked at the relationship between samples using principal component analysis and unsupervised clustering. The first principle component comprised 54% of the variance and separated nuclear samples from cytoplasmic samples. The second principal component that comprised 11% of the variance in the data was related to batch effect, although interestingly this primarily affected the variance of nuclear samples. This batch effect was corrected for in subsequent statistical analysis. Expression of miRNA from distinct subcellular components clustered together without any obvious differences between miRNA from SGC and LGC (Fig. 3a). We next examined expression of miRNA using a generalized linear model (see Methods) comparing all four groups (SGC nuclei, LGC nuclei, SGC cytoplasm, and LGC cytoplasm). While most miRNA that showed differences in expression between the nucleus and cytoplasm were common between SGC and LGC, several miRNA differed in their subcellular localization between granulosa cells from the two stages of follicular development. Further examination of nuclear miRNA revealed seven differentially expressed between SGC and LGC (Fig. 3b).

We then ranked miRNA based on their fold change between the nucleus and cytoplasm (Supplemental Tables 1 and 2). SGC showed more significantly different miRNAs between these subcellular compartments than LGC (101 versus 83, respectively).

**Fig. 4:**
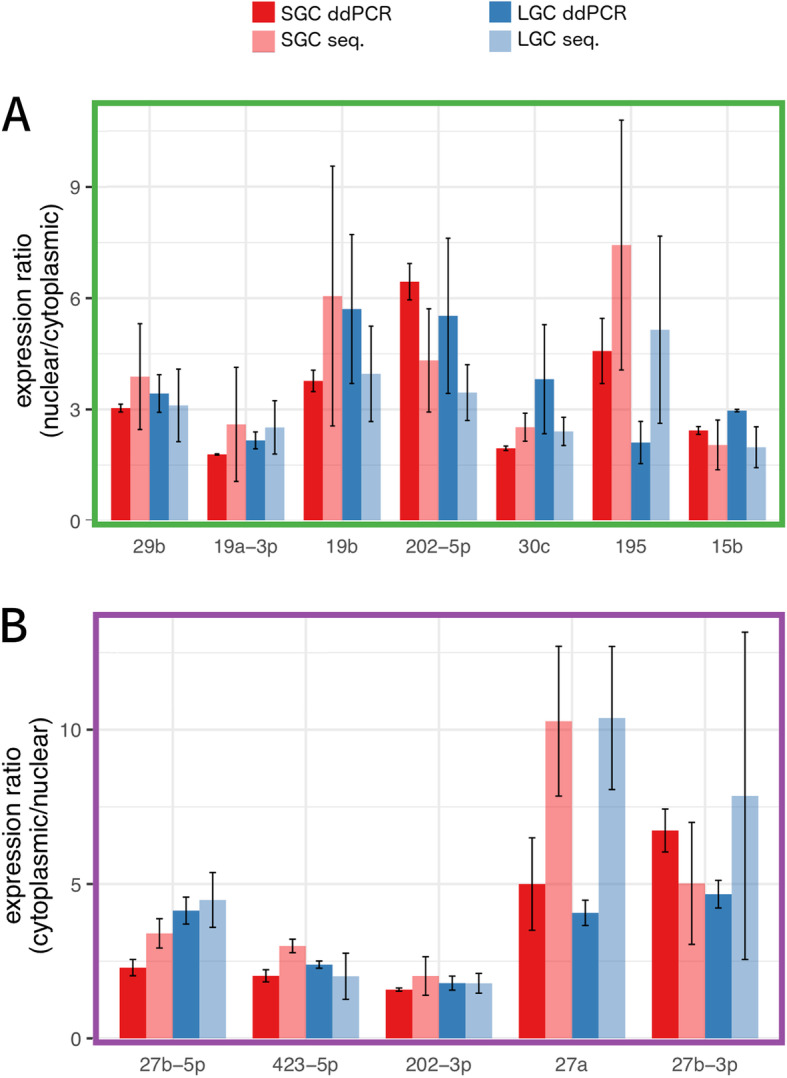
MicroRNA enrichment confirmed by digital drop RT-qPCR (ddPCR) in the **a** nucleus and **b** cytoplasm of SGC (red) and LGC (blue). Data represents the mean ratio of expression according to RNA sequencing (light bars) and ddPCR (dark bars) ± standard deviation

To confirm our sequencing results, we analyzed expression of 12 miRNA by digital droplet RT-PCR in our fractionated samples. All analyzed miRNA showed identical enrichment, either cytoplasmic or nuclear, and a significant correlation of expression ratios (*R* = 0.50, *P* = 0.013) between the two technologies (Fig. 4).

### Some nuclear miRNAs are predicted to target promoter regions

With specific miRNAs observed to be enriched in the nucleus of granulosa cells and previous work demonstrating that small RNA in the nucleus can regulate transcription, we examined the potential targeting of these nuclear miRNAs to genomic promoter regions. Because of a dearth of annotation for pig promoter regions, we used the fact that mature miRNAs are highly homologous [13] and looked first at binding sites in human promoter regions that were then mapped to homologous regions in the pig. Our analysis revealed 417 potential binding sites at the promoter regions across the human genome, of which 55 had identical matches to homologous regions in the pig genome (Supplemental Table 3 and Supplemental File 1). We then used all putative targets to conduct a gene set analysis [35] to check whether these miRNA were targeting common pathways. DNA-binding genes that positively regulate patterning, development and locomotion were all significantly enriched (Table 1).

**Table 1:** Gene set enrichment results of predicted miRNA promoter targets

Gene set ID	Gene set description	*P* Value	Adj. *P* value
GO:0045893	positive regulation of transcription, DNA-templated	0.00006	0.018
GO:0045595	regulation of cell differentiation	0.00027	0.035
GO:0022008	neurogenesis	0.00039	0.035
GO:0035295	tube development	0.00045	0.035
GO:0051960	regulation of nervous system development	0.00143	0.077
GO:0055123	digestive system development	0.00147	0.077
GO:0010648	negative regulation of cell communication	0.00276	0.090
GO:0051093	negative regulation of developmental process	0.00277	0.090
GO:0023057	negative regulation of signaling	0.00283	0.090
GO:0051094	positive regulation of developmental process	0.00324	0.090

### Putative novel miRNA identified in pig granulosa cells

**Fig. 5:**
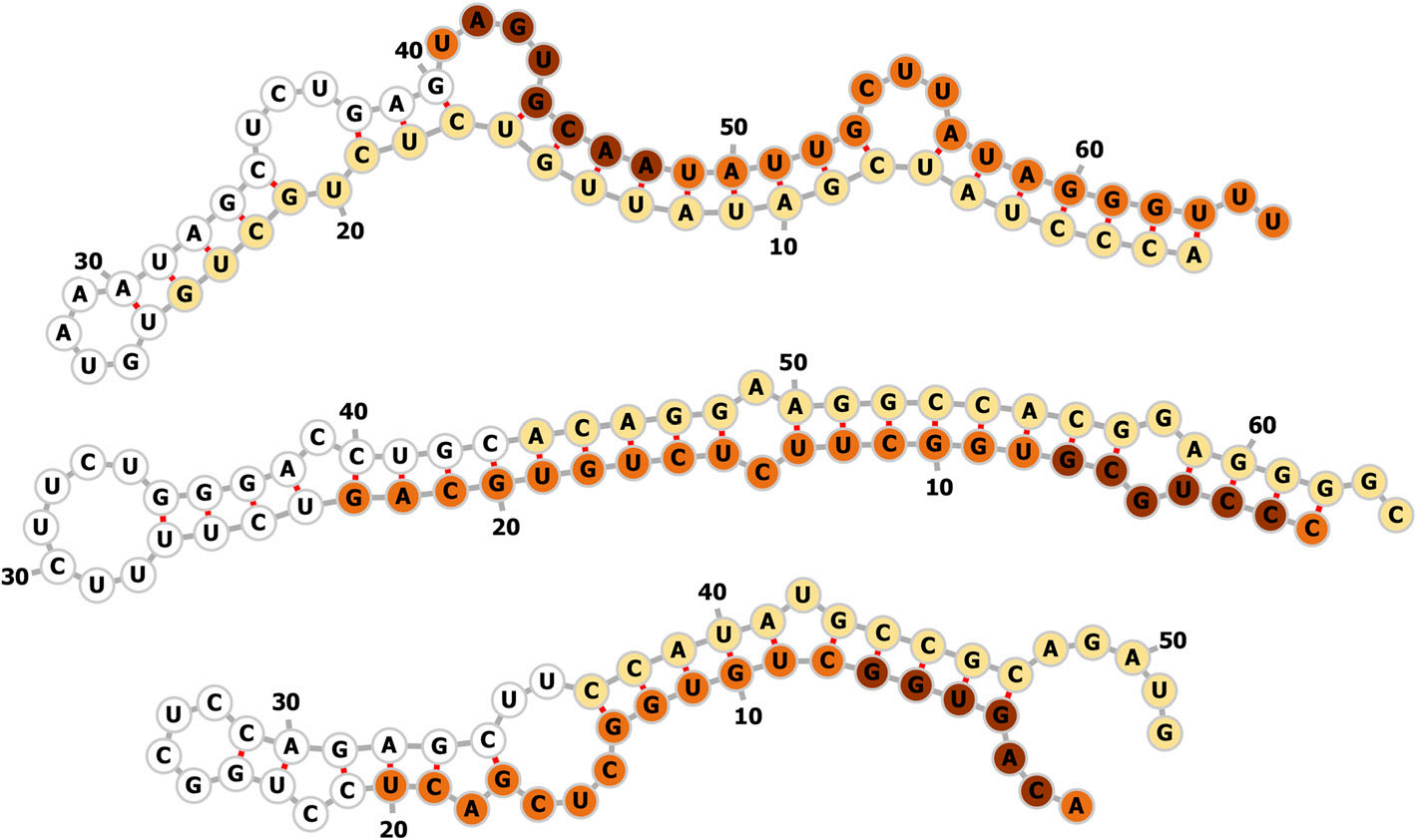
Predicted folding structure for putative novel pig miRNA stem loop precursors. Primary mature miRNA is shown in orange with the seed sequence in brown. Star sequences are shown in yellow. **Top:** ssc-mir-chr12 has the same seed sequence as hsa-miR-130a-3p and maps to the livestock (e.g. goat, horse, cow, sheep) miRNA miR-454. **Middle:** ssc-mir-chr2 has the same seed sequence as hsa-miR-3193 and very little homology with known sequences. **Bottom:** ssc-mir-chr13 has the same seed sequence as hsa-miR-5693 and matched several ESTs derived from the pig X chromosome

We took advantage of the miRDeep2 algorithm to predict novel miRNA based on precursor structure and read depth [12]. Using all reads from all groups, 39 miRNA were predicted with a score above 10, of which 34 ± 2 were estimated to be true positives. From this list, we checked ten with the highest number of mature read counts (all greater than 500; median of 2841). Six sequences aligned to other RNA species (e.g. Y RNA, rRNA, tRNA), while one appeared to be a single nucleotide mismatch to ssc-miR-26a. The three remaining sequences shown in Fig. 5 likely represent novel pig miRNA based on structure and seed sequence similarity to known human miRNAs: hsa-miR-130a-3p, hsa-miR-3193 and hsa-miR-5693.

### Other small RNA species are also present in the cytoplasm and nucleus

As we had observed significant numbers of reads mapping to snoRNA and piRNA (Fig. 2b), we looked further into their subcellular distributions in granulosa cells. We found that piRNA were more abundant in the nucleus and showed a greater variation between SGC and LGC here than in the cytoplasm (data not shown). As expected, no reads mapped to piRNA found on the Y chromosome.

**Fig. 6:**
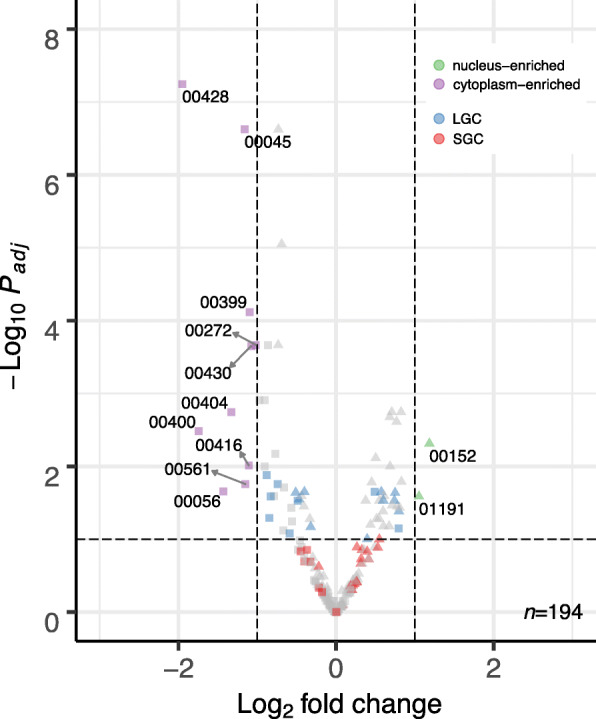
Volcano plot of mapped snoRNA showing expression log fold change relative to the nucleus and significance of the enrichment as given by the -log10 of the adjusted *p*-value. Each point represents an RFAM sequence with accession numbers in the form RFxxxxx, where triangles denote C/D family snoRNA and squares denote H/ACA family snoRNA. Red and blue points denote snoRNA exhibiting differential expression in only SGC or only LGC, respectively

Similarly, we looked at the distribution of snoRNA, which were also found throughout the cell. Most significantly different snoRNA were those found in the cytoplasm, made up exclusively of box H/ACA type snoRNA, while those found in the nucleus belonged to the box C/D class snoRNA (Fig. 6). One snoRNA, SNORD86 (accession RF00594), showed significant nuclear enrichment, but only in small granulosa cells.

## Discussion

Our expression profile in porcine granulosa cells compared small non-coding RNA at two stages of ovarian follicle development and looked at their distribution in the nucleus and cytoplasm. Read lengths were similarly distributed between all samples and mapping to specific RNA species followed an expected subcellular distribution. The large fraction of uncharacterized “miscellaneous reads” found in the cytoplasm (Fig. 2b) may be due to degraded RNA species. While most of these will be degraded (reviewed in [21]) recent evidence has suggested that some short RNAs have functional roles within the cell [9]. With continued investigation in this field, a much broader role for these small RNA is likely to be uncovered. We observed a substantial batch effect on nuclear miRNA and snoRNA, suggesting an unstable nuclear component that could be sensitive to processing. Our statistical models take this into account but it would be interesting to further investigate whether this source of variation is technical or biological in nature.

Broadly, miRNA were not substantially different between SGCs and LGCs, nor was the expression of cytoplasmic miRNAs. We did, however, identify seven nuclear miRNAs that were differentially expressed between SGC and LGC (Fig. 3b). Of note, these microRNA have been shown to play roles in granulosa cell function, including proliferation and apoptosis. The miR-182 cluster has been shown to promote proliferation in bovine granulosa cells by directly targeting the forkhead box protein O1 (FOXO1) [15]. Our data shows higher levels of miR-182 in LGC, consistent with a proliferative phenotype. Conversely, miR-181a showed a decrease in expression from SGC to LGC. This miRNA was demonstrated to increase FOXO1 acetylation by targeting the deacetylase sirtuin 1, ultimately promoting granulosa cell apoptosis [65]. Another study found that this same miRNA targeted the activin receptor IIA (ACVR2A) in mouse granulosa cells and its expression promoted an anti-proliferative phenotype [66]. Interestingly, this group also found that whole-cell expression of miR-181a decreased during follicle development, which is in line with our findings. In rat ovaries, miR-122 has been shown to target the luteinizing hormone receptor [42] although we found no change in cytoplasmic levels of this miRNA during follicular growth. We did see a considerable increase in nuclear expression of miR-122 in LGC. This miRNA has been shown to regulate the processing of pre-miR-21 in hepatocellular carcinoma cells and promoted apoptosis [60]. Whether this same miRNA processing control is exerted in granulosa cells is intriguing, especially as we have recently identified miR-21 as playing an important role in cumulus-oocyte complex maturation [44]. MiR-126-5p showed a similar trend as miR-122, namely increased nuclear expression in LGCs. This miRNA has been shown to target the follicle-stimulating receptor (FSHR) in pig granulosa cells, suppress cytochrome 19A1 (aromatase) expression, and promote apoptosis [8, 32]. Aromatase, and consequently estradiol production, is also negatively regulated by miR-378, as we have previously reported [45, 57, 64]. Other aspects of follicle development have also been reported to be regulated by miR-378 [23, 54, 59], although to our knowledge this is the first time this analysis has been undertaken at a subcellular level. Here it appears that the known decrease in miR-378 in porcine granulosa cells taking place during follicle growth is derived primarily from changes in nuclear expression. Further studies should reveal how nuclear miR-378 regulates granulosa function. Less well-studied, nuclear expression of miR-142-5p and miR-192 was higher in LGC; these two miRNA have been found to be increased in patients with polycystic ovarian syndrome and diminished ovarian reserve, respectively [33, 63], but their role in ovarian function is not known. Taken together, our results are supported by other studies of granulosa cell miRNAs. We did observe very significant differences in miRNA expression between the nucleus and cytoplasm when considering both groups together; a larger sample size may have had sufficient power to detect additional differentially expressed miRNA. Combining samples of both sizes (LGC and SGC) also allowed us to increase the reads available to miRDeep2 to predict novel miRNA. We identified three high-confidence novel miRNAs present in pig granulosa cells on chromosomes 2, 12, and 13. None of these showed a strong bias towards either subcellular compartment (data not shown), but as part of a complex miRNA network [13, 24] they are undoubtedly important in regulating granulosa cell behaviour and warrant future investigation into their precise functions. Cytoplasm-enriched miRNAs included well known, abundant members like let-7 isomirs and miR-27b [1]. No changes in let-7 family expression associated with atresia in the pig follicle [4, 68] were found between SGC and LGC groups. We also did not observe any pattern in the sequences of mature miRNA between nucleus- and cytoplasm-enriched (data not shown). While a hexanucleotide motif has been shown to be important for miR-29b [22], this has not been observed by others [5, 34], which agrees with our results presented here. Of note, we did observe strong nuclear enrichment of miR-29b in both SGC and LGC (Fig. 4a; Supplemental Tables 1 and 2). Other lines of evidence have suggested that the subcellular distribution of miRNA is dependent on target abundance, and miRNA are shuttled to the appropriate cellular compartment as needed [49]. Argonaute-loaded miRNAs have been found to target nuclear RNA species, including introns and lncRNAs, which supports the idea that post-transcriptional regulation occurs in the nucleus and cytoplasm [14, 61]. The complete list of determinants for nuclear import of miRNA, however, remain to be elucidated.

In addition to post-transcriptional repression, a more recently appreciated role of nuclear miRNA is RNA activation (RNAa) whereby these small RNA can be recruited to complex nucleic acid scaffolds to recruit and stabilize transcriptional co-factors [19]. Gene ontology analysis of promoter regions targeted by nucleus-enriched miRNA revealed enrichment of direct DNA-binding proteins. Parallel mechanisms involving either small RNA or DNA-binding proteins have been shown to be responsible for chromatin modifications in yeast [25]. While no reports in mammalian cells link these two phenomena, the involvement of small RNA in heterochromatin is thought to be conserved (reviewed in [16]) and an autoregulatory loop between DNA-binding proteins and miRNA has been observed [58]. The miRNA observed in our study that bind promoters of DNA-binding proteins could serve as redundant or feed-forward mechanisms to regulate transcription in granulosa cells during the final stages of follicle maturation. Several genes were associated with developmental processes, including the ubiquitously expressed upstream binding transcription factor (UBTF), a putative transcriptional target of let-7e that has been shown to regulate transcription to maintain homeostasis during cell growth [53]. A study of human follicles showed higher oocyte expression levels of *UBTF* in antral compared with secondary follicles [67], consistent with a role in cell proliferation. A second predicted developmental target of let-7e is reticulon (RTN)4. While no in-depth studies exist for this family of endoplasmic reticulum-associated proteins in the ovary, other reticulon genes and receptor-like proteins in the follicle have been correlated to better embryo development in vitro [43]. In mouse granulosa cells, RTN4 was a direct target of AKT1, itself a critically important regulator of cell growth and organized locomotion [10]. Roles for let-7 family miRNAs other than let-7e have been demonstrated in the pig ovarian follicle [4, 68]. Given their high sequence homology, it is likely that some degree of target redundancy exists between members of this family. The steroid receptor RNA activator (SRA)1 gene was a predicted target of miR-339-5p. Knockdown of SRA1 in ovarian endometriosis increased estrogen receptor (ER)-alpha expression at the expense of ER-beta [36]. Although SRA1 is expressed in the ovary, neither the long noncoding RNA nor the protein it codes for have been studied in granulosa cells. In ovarian cancer cells, miR-339-5p inhibits proliferation [38], although its role in normal follicle growth has not been established. These putatively targeted genes are also involved with biological processes that include differentiation and signaling in neurogenesis. Given the role of neurotrophins in ovarian development [7, 27, 37], our results suggest miRNA-mediated RNAa in granulosa cells may be another mechanism by which these small RNA regulate follicle growth.

We also profiled the snoRNA and piRNA that were captured by our sequencing data. The most studied function of mammalian piRNAs is the maintenance of genomic integrity in gametes [52]. In spite of this, changes in piRNA populations have been implicated in ovarian cancers and low ovarian follicle reserves [2, 6, 56]. The repetitive nature of piRNA make mapping short reads to the genome difficult, but chromosomal piRNA expression appeared to be consistent between subcellular compartments. We also observed higher levels of piRNA and a larger difference between SGC and LGC in the nucleus. As the primary transposon silencing activity of piRNA takes place in the cytoplasm of gametes, our results could suggest nuclear functions like transcriptional gene silencing predominate in granulosa cells [50]. A similar pattern of nuclear piRNA expression has been observed in cancer cells [5], although discerning whether these piRNA represent nascent transcripts originating in the nucleus or mature sequencing functioning there requires further study.

A similarly-limited focus has been placed on the role of snoRNA in the ovary. As both intact structures, and as a source of short regulatory RNAs, the role of snoRNA in cells continues to expand [11]. A study looking at the box H/ACA snoRNA U17/SNORA73 found that it inhibits hypoxia-upregulated mitochondrial movement regulator (HUMMR) during cholesterol trafficking [26]. Indeed, the authors find a reciprocal relationship between decreasing SNORA73 and increasing HUMMR during mouse ovary development; SNORA73 inhibition increased levels of ovarian pregnenolone and progesterone [26]. This role in steroid synthesis is supported by our finding that SNORA73 is one of the most significantly cytoplasm-enriched snoRNA in granulosa cells (Fig. 5; accession RF00045). SnoRNAs may yet prove to be play an important role in ovarian function.

Overall, we have provided a survey of the subcellular small RNAome in pig preovulatory follicle granulosa cells. The data used for this analysis is freely available at github.com/derektoms/s3RNA, and we anticipate that it will be a valuable resource for others studying the role of small RNA in the pig ovary. Increasing granularity in small RNA profiling, including from single cells [18], will continue to increase our understanding of the complex role these molecules play in ovarian granulosa cells.

## Methods

### Granulosa cell isolation

Details of the isolation and culture of granulosa cells has been described previously [46]. Briefly, porcine ovaries were removed from gilts at a local slaughterhouse and returned to the laboratory within 1.5 h in sterile 1x PBS at 22 °C. The ovaries were rinsed at least three times with 1x PBS. Granulosa cells were removed from small-sized follicles (1–3 mm in diameter) and large-sized follicles (3–6 mm in diameter) with a 20-gauge needle fixed to a 20-ml disposable syringe. Each sample represents a biologically distinct pool of granulosa cells from follicles of multiple ovaries that were subsequently fractioned.

### Preparation of cytosolic and nuclear fractions

The separation of cytoplasmic and nuclear was performed in accordance with the manufacture’s guidelines (Nuclei EZ Prep Nuclei Isolation Kit, Product Code: NUC-101). Briefly, fresh granulosa cells were transferred into a separate 15-ml centrifuge tube and centrifuged at 500 x g for 5 min at 4 °C. Cells were kept on ice and washed with 10 ml ice cold PBS twice. Nuclei EZ lysis buffer and brief vortexing was used to lyse cells; nuclei were collected by centrifugation at 500 x g for 5 min at 4 °C. The cytoplasmic fraction was selected from the upper 250 ul and the nuclear fraction from the lower 45 ul, discarding an intermediate layer of around 200 ul with the intention of controlling cross-contamination. Cold Nuclei EZ storage buffer was added to nuclear fraction, followed by further vortexing and trituration to help break up clumps of nuclei.

Nuclei for RNA and protein analysis were immediately frozen at − 80 °C in Nuclei EZ storage buffer. For assessing morphological quality and yield, nuclei were placed on a glass slide and microscopically examined. The nuclei were stained with either Hoechst 33342 (10 ng μl^− 1^), or 0.2% (vol/vol) trypan blue solution in PBS to confirm the purity.

### Western blotting analysis and antibodies

The cell nuclear pellets were lysed by using the Immunoprecipitation & Western Blotting RIPA Lysis Buffer (Cat 20–188, Sigma USA). Western blots were carried out using 4-20% Mini-PROTEAN® TGX Stain-Free™ Precast Gels (Cat. 4568095, Bio-Rad, Hercules, CA, USA) loaded with 50 to 100 μg of lysate and subsequently transferred to 0.45 μm Immun-Blot® low fluorescence PVDF Membrane (Cat. 1620260, Bio-Rad) using a Trans-Blot Turbo apparatus (Bio-Rad). Gels were activated by ultraviolet (UV) exposure for 2 min using a Bio-Rad ChemiDoc MP imager and total protein for each loading well were assessed. After protein transfer, membranes were imaged for stain-Free staining and total protein was quantified using Image-Lab 4.1 software (Bio-Rad). After transfer, the gels were stained with Bio-Safe™ Coomassie Stain (Cat. 1610786, BioRad) for 15 to 60 min and quickly de-stained in water to remove nonspecific staining. The membranes were then imaged, and total leftover proteins were measured using Image-Lab 4.1. The transferred membranes were blocked with 5% (w/v) nonfat milk in TBST for 1 h and then were cut into two separate pieces according to the different sizes. The upper blot (bigger than 50 kDa) was incubated with the nuclear envelope marker Anti-Lamin B1 antibody (1:4,000, Abcam, ab16048, Rabbit polyclonal, Cambridge, MA) followed by incubation with secondary antibody (anti-Rabbit IgG horseradish peroxidase, 1:4,000; Cell Signaling Technology, Danvers, MA, USA) for 1 h at room temperature; The lower blots were incubated with the Anti-GAPDH antibody [6C5] (1:4,000, abcam, ab8245, Mouse monoclonal, 1:2,000, MA, USA) for 1 h, following by incubation with secondary antibody (anti-mouse IgG horseradish peroxidase, 1:4,000; Cell Signaling Technology, Danvers, MA, USA). Membranes were again washed with TBST three times for 10 min each, incubated with Clarity chemiluminescence substrate (Bio-Rad), and imaged on the ChemiDoc MP, and then the bands were detected and analyzed with ImageLab 4.1.

### RNA isolation and quality control

Total RNA Purification Kit (Norgen Biotek, Cat. 17200; Thorold, ON, Canada) was used to isolate total RNA from both cytoplasm and nuclear samples. Briefly, around 3 × 10^6^ granulosa nucleus or 100 μl cytoplasm lysate were mixed with 350 μL of Buffer RL, then vortexed for up to 60 s to ensure the mixture becomes transparent. 200 μL of 96–100% ethanol was added into to the lysate following by vortexing for 10–30 s. Lysate with ethanol was added onto the column and centrifuge for 1 min at 3,500 x g to allow the entire lysate to pass through the column. RNase-Free DNase I Kit was used to minimize genomic DNA contamination in according with the manufacture’s On-Column DNA Removal Protocol (Norgen, Cat. 25710). After genomic DNA removal, the column was washed and centrifuge twice. 50 μL of elution solution was used to recover the total RNA. Quality and quantity of total RNA were determined using a NanoDrop ND-1000 Spectrophotometer at the Genomics Facility in the University of Guelph’s Advanced Analysis Centre. Using Agilent 2100 Bioanalyzer DNA and RNA chips, we verified that DNA concentration in the nuclear fraction was >100X that of the cytoplasmic fraction and that 28S ribosomal RNA peak fluorescence in the cytoplasmic fraction was >10X that of the nuclear fraction. These two ratios for the same samples imply good fractionation.

### Library preparation and sequencing

Small RNA library preparation and sequencing was performed by Personalized Genomics and Innovative Medicine in Toronto, Canada.

Briefly, small RNAs ranging from 18 to 52-nt were gel-purified and ligated to the 39 adaptor (59-pUCGUAUGCCGUCUUCUGCUUGidT-39; p, phosphate; idT, inverted deoxythymidine) and 59 adaptors (59-GUUCAGAGUUCUACAGUCCGACGAUC-39). Ligation products were gel-purified, reverse transcribed, and amplified using Illumina’s sRNA primer set (59-CAAGCAGAAGACG GCATACGA-39; 59-AATGATACGGCGACCACCGA39). Samples were sequenced on an Illumina 1G Genome Analyzer.

### Bioinformatic analysis

#### Sequence processing

Samples were sequenced on the rapid run mode of Illumina HiSeq 2500 sequencing platform. Base calling (BCL) files from sequencer were converted to the FASTQ files using bcl2fastq2-v2.17.1.14 tool. The raw reads generated were single-end and of length 51-nt. Raw reads quality in FASTQ format was assessed using FastQC tool v0.11.0 [62] and identified over-represented sequences (e.g. small RNA adapter and RNA PCR primer sequences). Small RNA sequencing data analysis conducted using miRDeep2 package [12]. Mapper module from miRDeep2 package requires raw reads (FASTQ format) as the input. Mapper module contains the data preprocessing functionalities that involves the conversion of FASTQ to FASTA, RNA to DNA, remove non-ATGCUN/atgcun bases, trimming Illumina small RNA adapter sequences (TGGAATTCTCGGGTGCCAAGG) and RNA primer sequences, filtering low-quality reads with < 18 nt length and collapsing the identical reads to remove redundancy. To quantify the number of reads independently mapped to pig mature and hairpin miRNAs sequences from miRBase v21 [17, 29, 30], Ensembl CDS, other classes of non-coding RNAs [lincRNA, scaRNA, snoRNAs, snRNAs, rRNAs, sRNAs, tRNAs, mitochondrial tRNAs, mitochondrial rRNAs, miscRNAs; piRNAs] and finally to pig genome (http://igenomes.illumina.com.s3-website-us-east-1.amazonaws.com/Sus_scrofa/UCSC/susScr3) using the mapper module of miRDeep2 package with the following parameters (−e -h -i -j -k TGGAATTCTCGGGTGCCAAGG -l 18 -m -p –s –t –v). Followed by alignment of the collapsed reads against the pig reference genome (susScr3) using bowtie to generate mapped output in ARF format and collapsed reads in fasta format. The core miRDeep2 module is used to detect known and novel small RNAs, that involves the following input files, the mapped output (ARF), collapsed reads (FASTA), pig reference genome (FASTA) and the miRBase v21 mature and hairpin reference sequences (FASTA). In-house perl scripts were written to detect and quantify all types of isomiRs, which were treated as reads for a single mature miRNA.

#### Expression analysis

The R package *DESeq2* (v 1.22. 2[40];) was used to detect differential expression in small RNA-Seq data, specifically miRNA and snoRNA. Matrices of read counts (small RNA vs samples) were analyzed by DESeq2 using two different models, one based on two variables (GC size and subcellular localization) and the other on a single “group” variable combining size and localization (e.g. “SGC-nuclear”). Both models accounted for batch effects. Statistically significant RNAs were be filtered using an adjusted *p*-value < 0.1 based on the negative binomial distribution.

For hierarchical cluster analysis, data was transformed using a regularized log method [40] before unsupervised clustering was performed with the *pheatmap* package based on the Euclidean distances between transformed expression values using a complete linkage method (*v 1.0.1 2*[28];). For other visualization and ranking of differentially expressed genes, shrinkage of the log fold change estimates (effect size) was performed using the *apeglm* method [69].

#### Prediction of miRNA binding to the promoter

Target genes were predicted using computational prediction software MicroPIR2 (biotech4/2). This software contains a database with over 80 million miRNA predicted targets in the promoter sequences of the human genome. MicroRNA targets were searched by miRNA name, with an average p-value ≤0.05, average conservation score ≥ 0.85, and a maximum number of four unpaired nucleotides. The binding pattern and sequence was observed with consideration towards near perfect matching in the seed region. Ensembl, a genetic database, was then used to determine if the predicted human promoter target sequence is conserved in *Sus scrofa* by identifying the sequence within the genome of *Sus scrofa* using the “BLAST” feature. The selected genes were then searched in the Ensembl *Sus scrofa* database to determine the target sequences’ relative location to the start codon, ATG in order to confirm the target sequence is indeed in the promoter region, which is ~ 1000 bp upstream of ATG and ~ 200 bp downstream of ATG.

## Supplementary Information

**Additional file 1.**

**Additional file 2.**

## Data Availability

Raw sequences have been deposited to https://www.ncbi.nlm.nih.gov/sra/PRJNA604978 and the project is available at https://www.ncbi.nlm.nih.gov/bioproject/PRJNA604978 Processed read count data for miRNA and snoRNA, and R code used for analysis can be accessed at github.com/derektoms/s3RNA

## Abbreviations

LGC: Large preovulatory (4–6 mm diameter) follicle-derived granulosa cells;
SGC: Small preovulatory (0.5–3 mm diameter) follicle-derived granulosa cells
ncRNA: Non-coding RNA
miRNA: MicroRNA
piRNA: PIWI-interacting RNA
snoRNA: Small nucleolar RNA
