# Supplementary material for: Small RNA sequencing reveals distinct nuclear microRNAs in pig granulosa cells during ovarian follicle growth
Source: J Ovarian Res. 2021 Apr 20;14:54. doi: 10.1186/s13048-021-00802-3 (PMC8059229; doi:10.1186/s13048-021-00802-3)
Supplement: Supplementary file 1 — Additional file 1. [file 13048_2021_802_MOESM1_ESM.docx]

Small RNA sequencing reveals distinct nuclear microRNAs in granulosa cells during ovarian follicle growth

Derek Toms, Bo Pan, Yinshan Bai, Julang Li

Supplemental Information

***Supplemental Table 1.*** *MicroRNA subcellular expression profile of SGC.*

| miRNA | baseMean | log2FoldChange | lfcSE | stat | pvalue | padj |
| --- | --- | --- | --- | --- | --- | --- |
| **ssc-miR-3613** | 3.88E+00 | 3.51E+00 | 1.18E+00 | 2.97E+00 | 2.98E-03 | 9.86E-03 |
| **ssc-miR-29b** | 7.86E+00 | 2.75E+00 | 8.03E-01 | 3.42E+00 | 6.17E-04 | 2.38E-03 |
| **ssc-miR-1296-5p** | 2.54E+01 | 2.48E+00 | 4.37E-01 | 5.67E+00 | 1.42E-08 | 2.16E-07 |
| **ssc-miR-769-3p** | 1.23E+01 | 2.37E+00 | 5.54E-01 | 4.28E+00 | 1.91E-05 | 1.19E-04 |
| **ssc-miR-19a** | 1.99E+01 | 2.27E+00 | 4.60E-01 | 4.93E+00 | 8.26E-07 | 8.76E-06 |
| **ssc-miR-505** | 1.37E+01 | 2.16E+00 | 5.07E-01 | 4.26E+00 | 2.06E-05 | 1.25E-04 |
| **ssc-miR-19b** | 1.01E+02 | 2.05E+00 | 3.37E-01 | 6.08E+00 | 1.18E-09 | 2.28E-08 |
| **ssc-miR-769-5p** | 2.28E+02 | 1.95E+00 | 3.29E-01 | 5.94E+00 | 2.93E-09 | 5.17E-08 |
| **ssc-miR-30b-5p** | 7.34E+01 | 1.92E+00 | 2.87E-01 | 6.71E+00 | 1.93E-11 | 8.17E-10 |
| **ssc-miR-34a** | 3.11E+01 | 1.84E+00 | 4.16E-01 | 4.41E+00 | 1.01E-05 | 7.15E-05 |
| **ssc-miR-15b** | 3.43E+02 | 1.79E+00 | 2.42E-01 | 7.37E+00 | 1.70E-13 | 1.66E-11 |
| **ssc-miR-339-5p** | 6.07E+01 | 1.57E+00 | 3.28E-01 | 4.79E+00 | 1.66E-06 | 1.53E-05 |
| **ssc-miR-324** | 9.64E+00 | 1.52E+00 | 5.58E-01 | 2.73E+00 | 6.41E-03 | 1.92E-02 |
| **ssc-miR-339** | 4.62E+01 | 1.51E+00 | 3.34E-01 | 4.52E+00 | 6.20E-06 | 4.70E-05 |
| **ssc-miR-1249** | 7.80E+00 | 1.46E+00 | 7.31E-01 | 2.00E+00 | 4.55E-02 | 9.75E-02 |
| **ssc-miR-374b-5p** | 1.39E+01 | 1.46E+00 | 4.78E-01 | 3.04E+00 | 2.34E-03 | 8.14E-03 |
| **ssc-miR-331-3p** | 1.49E+01 | 1.44E+00 | 5.34E-01 | 2.70E+00 | 7.02E-03 | 2.01E-02 |
| **ssc-let-7d-3p** | 1.10E+02 | 1.42E+00 | 3.14E-01 | 4.53E+00 | 5.94E-06 | 4.66E-05 |
| **ssc-miR-202-5p** | 6.71E+02 | 1.34E+00 | 1.95E-01 | 6.86E+00 | 6.85E-12 | 3.63E-10 |
| **ssc-miR-425-3p** | 7.41E+00 | 1.34E+00 | 6.25E-01 | 2.14E+00 | 3.21E-02 | 7.33E-02 |
| **ssc-miR-4334-3p** | 2.56E+01 | 1.23E+00 | 3.88E-01 | 3.16E+00 | 1.59E-03 | 5.73E-03 |
| **ssc-miR-17-5p** | 1.85E+02 | 1.21E+00 | 2.29E-01 | 5.29E+00 | 1.25E-07 | 1.56E-06 |
| **ssc-miR-18a** | 1.31E+01 | 1.19E+00 | 4.69E-01 | 2.54E+00 | 1.11E-02 | 2.94E-02 |
| **ssc-miR-chr3_17659** | 9.42E+00 | 1.17E+00 | 5.78E-01 | 2.03E+00 | 4.29E-02 | 9.37E-02 |
| **ssc-miR-874** | 1.27E+02 | 1.15E+00 | 3.18E-01 | 3.63E+00 | 2.84E-04 | 1.31E-03 |
| **ssc-miR-7134-5p** | 5.16E+01 | 1.11E+00 | 2.91E-01 | 3.81E+00 | 1.41E-04 | 7.31E-04 |
| **ssc-miR-140-3p** | 1.22E+03 | 1.11E+00 | 2.16E-01 | 5.12E+00 | 3.12E-07 | 3.67E-06 |
| **ssc-miR-195** | 6.46E+02 | 1.09E+00 | 2.58E-01 | 4.21E+00 | 2.51E-05 | 1.44E-04 |
| **ssc-miR-181d-5p** | 1.12E+02 | 1.00E+00 | 2.91E-01 | 3.44E+00 | 5.86E-04 | 2.34E-03 |
| **ssc-miR-24-3p** | 2.22E+02 | 9.83E-01 | 2.25E-01 | 4.36E+00 | 1.28E-05 | 8.75E-05 |
| **ssc-miR-20a** | 1.25E+02 | 9.63E-01 | 2.44E-01 | 3.95E+00 | 7.90E-05 | 4.26E-04 |
| **ssc-miR-30c-5p** | 3.54E+02 | 9.40E-01 | 2.62E-01 | 3.58E+00 | 3.39E-04 | 1.53E-03 |
| **ssc-miR-210** | 1.07E+02 | 9.39E-01 | 2.57E-01 | 3.65E+00 | 2.57E-04 | 1.24E-03 |
| **ssc-miR-190a** | 1.75E+01 | 9.36E-01 | 4.72E-01 | 1.98E+00 | 4.76E-02 | 9.99E-02 |
| **ssc-miR-185** | 1.43E+01 | 8.89E-01 | 4.37E-01 | 2.04E+00 | 4.17E-02 | 9.21E-02 |
| **ssc-miR-335** | 5.91E+01 | 8.71E-01 | 2.73E-01 | 3.19E+00 | 1.43E-03 | 5.23E-03 |
| **ssc-miR-1306-5p** | 2.32E+01 | 8.55E-01 | 3.68E-01 | 2.32E+00 | 2.01E-02 | 4.84E-02 |
| **ssc-miR-374a-5p** | 3.52E+01 | 8.29E-01 | 3.21E-01 | 2.58E+00 | 9.91E-03 | 2.69E-02 |
| **ssc-miR-30d** | 2.28E+03 | 8.22E-01 | 3.08E-01 | 2.67E+00 | 7.68E-03 | 2.17E-02 |
| **ssc-miR-574** | 7.72E+01 | 7.40E-01 | 3.14E-01 | 2.35E+00 | 1.86E-02 | 4.54E-02 |
| **ssc-miR-30e-5p** | 1.36E+03 | 6.83E-01 | 2.52E-01 | 2.72E+00 | 6.62E-03 | 1.95E-02 |
| **ssc-miR-7134-3p** | 1.58E+02 | 6.53E-01 | 2.46E-01 | 2.65E+00 | 7.99E-03 | 2.23E-02 |
| **ssc-miR-378** | 1.96E+02 | 6.52E-01 | 2.28E-01 | 2.86E+00 | 4.23E-03 | 1.32E-02 |
| **ssc-miR-340** | 1.46E+02 | 6.25E-01 | 2.83E-01 | 2.21E+00 | 2.69E-02 | 6.21E-02 |
| **ssc-miR-2320-5p** | 7.17E+01 | 6.01E-01 | 2.66E-01 | 2.26E+00 | 2.39E-02 | 5.70E-02 |
| **ssc-miR-23b** | 6.31E+02 | 5.26E-01 | 2.04E-01 | 2.58E+00 | 9.88E-03 | 2.69E-02 |
| **ssc-miR-1307** | 1.86E+02 | 5.20E-01 | 2.32E-01 | 2.25E+00 | 2.47E-02 | 5.82E-02 |
| **ssc-miR-676-3p** | 3.10E+02 | -4.90E-01 | 1.93E-01 | -2.53E+00 | 1.13E-02 | 2.95E-02 |
| **ssc-miR-146a-5p** | 1.65E+02 | -5.53E-01 | 2.24E-01 | -2.47E+00 | 1.36E-02 | 3.43E-02 |
| **ssc-miR-92b-3p** | 3.28E+03 | -5.72E-01 | 2.85E-01 | -2.00E+00 | 4.51E-02 | 9.75E-02 |
| **ssc-miR-184** | 1.20E+03 | -5.75E-01 | 2.36E-01 | -2.44E+00 | 1.49E-02 | 3.71E-02 |
| **ssc-miR-16** | 3.63E+03 | -6.10E-01 | 2.15E-01 | -2.83E+00 | 4.65E-03 | 1.43E-02 |
| **ssc-miR-28-3p** | 2.16E+03 | -6.65E-01 | 2.31E-01 | -2.88E+00 | 4.01E-03 | 1.27E-02 |
| **ssc-miR-100** | 4.71E+02 | -7.13E-01 | 2.08E-01 | -3.43E+00 | 6.02E-04 | 2.36E-03 |
| **ssc-let-7f** | 4.06E+04 | -7.44E-01 | 1.98E-01 | -3.76E+00 | 1.69E-04 | 8.35E-04 |
| **ssc-miR-7139-5p** | 1.54E+02 | -7.69E-01 | 2.85E-01 | -2.70E+00 | 6.96E-03 | 2.01E-02 |
| **ssc-miR-1343** | 2.62E+02 | -7.74E-01 | 2.53E-01 | -3.05E+00 | 2.27E-03 | 8.02E-03 |
| **ssc-miR-132** | 5.86E+01 | -7.98E-01 | 3.18E-01 | -2.51E+00 | 1.22E-02 | 3.13E-02 |
| **ssc-miR-22-3p** | 1.55E+03 | -8.16E-01 | 2.69E-01 | -3.03E+00 | 2.42E-03 | 8.27E-03 |
| **ssc-miR-26a** | 5.25E+03 | -8.18E-01 | 2.17E-01 | -3.76E+00 | 1.68E-04 | 8.35E-04 |
| **ssc-let-7a** | 3.60E+04 | -8.55E-01 | 2.02E-01 | -4.23E+00 | 2.38E-05 | 1.40E-04 |
| **ssc-miR-155-5p** | 4.29E+01 | -9.24E-01 | 3.89E-01 | -2.38E+00 | 1.75E-02 | 4.31E-02 |
| **ssc-miR-744** | 4.16E+02 | -9.25E-01 | 2.55E-01 | -3.63E+00 | 2.81E-04 | 1.31E-03 |
| **ssc-miR-99a** | 5.39E+02 | -9.27E-01 | 2.35E-01 | -3.95E+00 | 7.71E-05 | 4.26E-04 |
| **ssc-miR-486** | 8.97E+01 | -9.42E-01 | 2.89E-01 | -3.27E+00 | 1.09E-03 | 4.06E-03 |
| **ssc-miR-27b-3p** | 1.45E+04 | -9.50E-01 | 1.94E-01 | -4.90E+00 | 9.77E-07 | 9.86E-06 |
| **ssc-miR-1468** | 1.45E+02 | -9.53E-01 | 3.28E-01 | -2.91E+00 | 3.64E-03 | 1.19E-02 |
| **ssc-miR-148a-3p** | 1.76E+03 | -9.75E-01 | 2.28E-01 | -4.29E+00 | 1.83E-05 | 1.17E-04 |
| **ssc-miR-30c-1-3p** | 9.63E+00 | -1.00E+00 | 5.06E-01 | -1.98E+00 | 4.75E-02 | 9.99E-02 |
| **ssc-miR-148a-5p** | 1.16E+01 | -1.02E+00 | 4.83E-01 | -2.10E+00 | 3.56E-02 | 8.03E-02 |
| **ssc-miR-2320-3p** | 2.20E+01 | -1.05E+00 | 3.66E-01 | -2.88E+00 | 3.95E-03 | 1.27E-02 |
| **ssc-let-7d-5p** | 1.11E+03 | -1.07E+00 | 2.42E-01 | -4.42E+00 | 9.98E-06 | 7.15E-05 |
| **ssc-miR-128** | 5.76E+02 | -1.11E+00 | 2.80E-01 | -3.94E+00 | 8.03E-05 | 4.26E-04 |
| **ssc-miR-215** | 4.23E+01 | -1.12E+00 | 3.20E-01 | -3.51E+00 | 4.57E-04 | 1.94E-03 |
| **ssc-let-7g** | 6.68E+03 | -1.15E+00 | 2.01E-01 | -5.71E+00 | 1.13E-08 | 1.84E-07 |
| **ssc-miR-143-3p** | 2.38E+03 | -1.19E+00 | 2.53E-01 | -4.69E+00 | 2.77E-06 | 2.44E-05 |
| **ssc-miR-28-5p** | 6.67E+01 | -1.19E+00 | 2.60E-01 | -4.57E+00 | 4.91E-06 | 4.00E-05 |
| **ssc-miR-2483** | 1.48E+01 | -1.20E+00 | 4.72E-01 | -2.54E+00 | 1.10E-02 | 2.94E-02 |
| **ssc-miR-126-5p** | 2.04E+02 | -1.20E+00 | 2.78E-01 | -4.33E+00 | 1.46E-05 | 9.70E-05 |
| **ssc-miR-151-5p** | 1.60E+03 | -1.24E+00 | 2.20E-01 | -5.63E+00 | 1.82E-08 | 2.57E-07 |
| **ssc-miR-130b** | 2.64E+01 | -1.26E+00 | 3.53E-01 | -3.56E+00 | 3.72E-04 | 1.65E-03 |
| **ssc-let-7e** | 3.46E+03 | -1.40E+00 | 2.19E-01 | -6.38E+00 | 1.83E-10 | 4.85E-09 |
| **ssc-miR-199a-3p** | 1.68E+01 | -1.42E+00 | 4.12E-01 | -3.45E+00 | 5.60E-04 | 2.28E-03 |
| **ssc-miR-199b-3p** | 1.66E+01 | -1.43E+00 | 4.14E-01 | -3.46E+00 | 5.39E-04 | 2.24E-03 |
| **ssc-miR-192** | 3.49E+03 | -1.45E+00 | 2.67E-01 | -5.44E+00 | 5.25E-08 | 6.95E-07 |
| **ssc-miR-130a** | 2.31E+02 | -1.47E+00 | 3.05E-01 | -4.81E+00 | 1.48E-06 | 1.43E-05 |
| **ssc-miR-126-3p** | 2.12E+01 | -1.50E+00 | 4.27E-01 | -3.52E+00 | 4.38E-04 | 1.89E-03 |
| **ssc-miR-421-3p** | 1.32E+02 | -1.51E+00 | 2.33E-01 | -6.47E+00 | 9.96E-11 | 3.52E-09 |
| **ssc-miR-27a** | 9.23E+01 | -1.56E+00 | 2.50E-01 | -6.24E+00 | 4.34E-10 | 1.02E-08 |
| **ssc-miR-7139-3p** | 6.55E+00 | -1.56E+00 | 6.23E-01 | -2.51E+00 | 1.22E-02 | 3.13E-02 |
| **ssc-miR-145-3p** | 5.25E+00 | -1.57E+00 | 7.68E-01 | -2.04E+00 | 4.10E-02 | 9.15E-02 |
| **ssc-miR-194a** | 2.65E+01 | -1.60E+00 | 4.68E-01 | -3.41E+00 | 6.50E-04 | 2.46E-03 |
| **ssc-miR-142-5p** | 4.29E+01 | -1.62E+00 | 3.51E-01 | -4.61E+00 | 4.09E-06 | 3.47E-05 |
| **ssc-miR-202-3p** | 5.75E+02 | -1.69E+00 | 2.31E-01 | -7.33E+00 | 2.34E-13 | 1.66E-11 |
| **ssc-miR-451** | 1.17E+01 | -1.80E+00 | 6.40E-01 | -2.81E+00 | 4.88E-03 | 1.48E-02 |
| **ssc-miR-664-5p** | 2.66E+01 | -1.80E+00 | 3.64E-01 | -4.95E+00 | 7.58E-07 | 8.45E-06 |
| **ssc-miR-122** | 8.82E+01 | -1.86E+00 | 2.90E-01 | -6.40E+00 | 1.59E-10 | 4.80E-09 |
| **ssc-miR-219a** | 8.09E+00 | -1.88E+00 | 6.27E-01 | -2.99E+00 | 2.77E-03 | 9.33E-03 |
| **ssc-miR-4332** | 1.42E+02 | -1.97E+00 | 8.83E-01 | -2.23E+00 | 2.58E-02 | 6.01E-02 |
| **ssc-miR-423-5p** | 3.74E+03 | -2.13E+00 | 3.43E-01 | -6.20E+00 | 5.50E-10 | 1.17E-08 |
| **ssc-miR-27b-5p** | 1.05E+02 | -3.28E+00 | 2.83E-01 | -1.16E+01 | 4.12E-31 | 8.73E-29 |

***Supplemental Table 2.*** *MicroRNA subcellular expression profile of LGC.*

| miRNA | baseMean | log2FoldChange | lfcSE | stat | pvalue | padj |
| --- | --- | --- | --- | --- | --- | --- |
| **ssc-miR-140-5p** | 1.25E+01 | 2.38E+00 | 6.16E-01 | 3.86E+00 | 1.12E-04 | 5.75E-04 |
| **ssc-miR-324** | 9.64E+00 | 2.34E+00 | 6.75E-01 | 3.47E+00 | 5.24E-04 | 2.30E-03 |
| **ssc-miR-671-5p** | 9.14E+00 | 2.25E+00 | 6.90E-01 | 3.27E+00 | 1.09E-03 | 4.18E-03 |
| **ssc-miR-29b** | 7.86E+00 | 2.18E+00 | 7.27E-01 | 3.00E+00 | 2.67E-03 | 9.33E-03 |
| **ssc-miR-331-3p** | 1.49E+01 | 2.13E+00 | 6.41E-01 | 3.33E+00 | 8.61E-04 | 3.60E-03 |
| **ssc-miR-19a** | 1.99E+01 | 1.95E+00 | 4.56E-01 | 4.28E+00 | 1.85E-05 | 1.11E-04 |
| **ssc-miR-1296-5p** | 2.54E+01 | 1.85E+00 | 4.17E-01 | 4.43E+00 | 9.56E-06 | 6.62E-05 |
| **ssc-miR-769-5p** | 2.28E+02 | 1.73E+00 | 3.31E-01 | 5.24E+00 | 1.59E-07 | 2.61E-06 |
| **ssc-miR-19b** | 1.01E+02 | 1.71E+00 | 3.40E-01 | 5.04E+00 | 4.54E-07 | 5.10E-06 |
| **ssc-miR-374b-5p** | 1.39E+01 | 1.71E+00 | 4.85E-01 | 3.53E+00 | 4.09E-04 | 1.89E-03 |
| **ssc-miR-339-5p** | 6.07E+01 | 1.71E+00 | 3.33E-01 | 5.13E+00 | 2.86E-07 | 3.95E-06 |
| **ssc-miR-339** | 4.62E+01 | 1.62E+00 | 3.43E-01 | 4.73E+00 | 2.28E-06 | 1.96E-05 |
| **ssc-miR-769-3p** | 1.23E+01 | 1.60E+00 | 5.33E-01 | 3.00E+00 | 2.70E-03 | 9.33E-03 |
| **ssc-miR-15b** | 3.43E+02 | 1.55E+00 | 2.41E-01 | 6.43E+00 | 1.27E-10 | 4.59E-09 |
| **ssc-miR-4334-3p** | 2.56E+01 | 1.51E+00 | 4.11E-01 | 3.67E+00 | 2.39E-04 | 1.16E-03 |
| **ssc-miR-34a** | 3.11E+01 | 1.45E+00 | 4.34E-01 | 3.34E+00 | 8.23E-04 | 3.53E-03 |
| **ssc-miR-30b-5p** | 7.34E+01 | 1.41E+00 | 2.83E-01 | 4.98E+00 | 6.39E-07 | 6.77E-06 |
| **ssc-miR-7134-5p** | 5.16E+01 | 1.38E+00 | 3.05E-01 | 4.52E+00 | 6.33E-06 | 4.95E-05 |
| **ssc-miR-190a** | 1.75E+01 | 1.35E+00 | 4.96E-01 | 2.71E+00 | 6.65E-03 | 2.14E-02 |
| **ssc-miR-374a-5p** | 3.52E+01 | 1.31E+00 | 3.24E-01 | 4.03E+00 | 5.67E-05 | 3.30E-04 |
| **ssc-miR-195** | 6.46E+02 | 1.28E+00 | 2.58E-01 | 4.97E+00 | 6.82E-07 | 6.82E-06 |
| **ssc-miR-328** | 1.25E+01 | 1.25E+00 | 4.84E-01 | 2.59E+00 | 9.56E-03 | 2.87E-02 |
| **ssc-miR-202-5p** | 6.71E+02 | 1.19E+00 | 1.96E-01 | 6.09E+00 | 1.12E-09 | 2.89E-08 |
| **ssc-miR-17-5p** | 1.85E+02 | 1.19E+00 | 2.34E-01 | 5.09E+00 | 3.53E-07 | 4.54E-06 |
| **ssc-miR-20a** | 1.25E+02 | 1.10E+00 | 2.51E-01 | 4.37E+00 | 1.27E-05 | 7.88E-05 |
| **ssc-miR-874** | 1.27E+02 | 1.06E+00 | 3.23E-01 | 3.29E+00 | 9.94E-04 | 3.89E-03 |
| **ssc-miR-24-3p** | 2.22E+02 | 1.03E+00 | 2.29E-01 | 4.50E+00 | 6.87E-06 | 5.15E-05 |
| **ssc-miR-361-5p** | 8.43E+01 | 9.88E-01 | 2.81E-01 | 3.52E+00 | 4.28E-04 | 1.93E-03 |
| **ssc-miR-30c-5p** | 3.54E+02 | 8.64E-01 | 2.62E-01 | 3.30E+00 | 9.82E-04 | 3.89E-03 |
| **ssc-miR-30e-5p** | 1.36E+03 | 7.76E-01 | 2.52E-01 | 3.08E+00 | 2.05E-03 | 7.40E-03 |
| **ssc-let-7d-3p** | 1.10E+02 | 6.82E-01 | 3.13E-01 | 2.18E+00 | 2.95E-02 | 7.38E-02 |
| **ssc-miR-186** | 5.06E+02 | 6.63E-01 | 2.37E-01 | 2.79E+00 | 5.23E-03 | 1.71E-02 |
| **ssc-miR-30d** | 2.28E+03 | 6.61E-01 | 3.08E-01 | 2.14E+00 | 3.22E-02 | 7.73E-02 |
| **ssc-miR-181d-5p** | 1.12E+02 | 6.34E-01 | 2.91E-01 | 2.18E+00 | 2.93E-02 | 7.38E-02 |
| **ssc-miR-210** | 1.07E+02 | 5.98E-01 | 2.61E-01 | 2.29E+00 | 2.20E-02 | 5.90E-02 |
| **ssc-miR-335** | 5.91E+01 | 5.68E-01 | 2.74E-01 | 2.07E+00 | 3.83E-02 | 8.95E-02 |
| **ssc-miR-140-3p** | 1.22E+03 | 5.38E-01 | 2.17E-01 | 2.48E+00 | 1.32E-02 | 3.89E-02 |
| **ssc-miR-1307** | 1.86E+02 | 4.83E-01 | 2.36E-01 | 2.05E+00 | 4.04E-02 | 9.19E-02 |
| **ssc-miR-23b** | 6.31E+02 | 4.66E-01 | 2.04E-01 | 2.28E+00 | 2.25E-02 | 5.96E-02 |
| **ssc-miR-191** | 6.94E+03 | 4.28E-01 | 1.81E-01 | 2.36E+00 | 1.82E-02 | 5.03E-02 |
| **ssc-miR-676-3p** | 3.10E+02 | -3.88E-01 | 1.93E-01 | -2.00E+00 | 4.51E-02 | 9.89E-02 |
| **ssc-let-7i** | 1.03E+04 | -4.27E-01 | 2.09E-01 | -2.04E+00 | 4.13E-02 | 9.29E-02 |
| **ssc-miR-181b** | 4.93E+02 | -4.35E-01 | 2.06E-01 | -2.11E+00 | 3.47E-02 | 8.22E-02 |
| **ssc-miR-26a** | 5.25E+03 | -4.72E-01 | 2.17E-01 | -2.17E+00 | 2.99E-02 | 7.38E-02 |
| **ssc-miR-22-3p** | 1.55E+03 | -5.42E-01 | 2.69E-01 | -2.02E+00 | 4.38E-02 | 9.74E-02 |
| **ssc-miR-151-3p** | 3.83E+03 | -5.52E-01 | 2.12E-01 | -2.61E+00 | 9.13E-03 | 2.79E-02 |
| **ssc-miR-7142-3p** | 5.57E+02 | -5.57E-01 | 2.28E-01 | -2.44E+00 | 1.46E-02 | 4.23E-02 |
| **ssc-miR-92b-3p** | 3.28E+03 | -5.69E-01 | 2.85E-01 | -2.00E+00 | 4.59E-02 | 9.96E-02 |
| **ssc-miR-107** | 4.97E+02 | -5.71E-01 | 2.52E-01 | -2.26E+00 | 2.36E-02 | 6.16E-02 |
| **ssc-miR-192** | 3.49E+03 | -5.77E-01 | 2.67E-01 | -2.16E+00 | 3.05E-02 | 7.42E-02 |
| **ssc-miR-99a** | 5.39E+02 | -6.62E-01 | 2.36E-01 | -2.80E+00 | 5.11E-03 | 1.70E-02 |
| **ssc-miR-215** | 4.23E+01 | -6.83E-01 | 3.12E-01 | -2.19E+00 | 2.86E-02 | 7.35E-02 |
| **ssc-miR-423-3p** | 1.62E+03 | -7.27E-01 | 2.75E-01 | -2.65E+00 | 8.14E-03 | 2.53E-02 |
| **ssc-miR-122** | 8.82E+01 | -7.44E-01 | 2.78E-01 | -2.68E+00 | 7.42E-03 | 2.34E-02 |
| **ssc-let-7f** | 4.06E+04 | -7.67E-01 | 1.98E-01 | -3.88E+00 | 1.06E-04 | 5.66E-04 |
| **ssc-miR-143-3p** | 2.38E+03 | -7.86E-01 | 2.53E-01 | -3.10E+00 | 1.92E-03 | 7.05E-03 |
| **ssc-let-7a** | 3.60E+04 | -8.00E-01 | 2.02E-01 | -3.96E+00 | 7.63E-05 | 4.29E-04 |
| **ssc-miR-27b-3p** | 1.45E+04 | -8.56E-01 | 1.94E-01 | -4.41E+00 | 1.04E-05 | 6.72E-05 |
| **ssc-miR-128** | 5.76E+02 | -8.84E-01 | 2.80E-01 | -3.15E+00 | 1.61E-03 | 6.04E-03 |
| **ssc-miR-2483** | 1.48E+01 | -9.10E-01 | 4.41E-01 | -2.06E+00 | 3.92E-02 | 9.04E-02 |
| **ssc-miR-132** | 5.86E+01 | -9.46E-01 | 3.23E-01 | -2.93E+00 | 3.40E-03 | 1.15E-02 |
| **ssc-miR-27a** | 9.23E+01 | -9.60E-01 | 2.48E-01 | -3.87E+00 | 1.07E-04 | 5.66E-04 |
| **ssc-let-7g** | 6.68E+03 | -9.80E-01 | 2.01E-01 | -4.89E+00 | 1.03E-06 | 9.80E-06 |
| **ssc-miR-127** | 4.41E+01 | -1.03E+00 | 4.38E-01 | -2.35E+00 | 1.86E-02 | 5.07E-02 |
| **ssc-miR-7139-5p** | 1.54E+02 | -1.04E+00 | 2.86E-01 | -3.62E+00 | 2.93E-04 | 1.39E-03 |
| **ssc-miR-1468** | 1.45E+02 | -1.08E+00 | 3.27E-01 | -3.29E+00 | 9.89E-04 | 3.89E-03 |
| **ssc-miR-151-5p** | 1.60E+03 | -1.11E+00 | 2.20E-01 | -5.05E+00 | 4.43E-07 | 5.10E-06 |
| **ssc-miR-100** | 4.71E+02 | -1.15E+00 | 2.09E-01 | -5.52E+00 | 3.38E-08 | 6.76E-07 |
| **ssc-miR-222** | 1.97E+01 | -1.17E+00 | 4.83E-01 | -2.42E+00 | 1.55E-02 | 4.44E-02 |
| **ssc-miR-28-5p** | 6.67E+01 | -1.19E+00 | 2.61E-01 | -4.57E+00 | 4.84E-06 | 3.96E-05 |
| **ssc-miR-28-3p** | 2.16E+03 | -1.20E+00 | 2.32E-01 | -5.20E+00 | 2.04E-07 | 3.06E-06 |
| **ssc-miR-744** | 4.16E+02 | -1.24E+00 | 2.55E-01 | -4.86E+00 | 1.19E-06 | 1.07E-05 |
| **ssc-let-7d-5p** | 1.11E+03 | -1.35E+00 | 2.42E-01 | -5.60E+00 | 2.15E-08 | 4.83E-07 |
| **ssc-miR-130a** | 2.31E+02 | -1.36E+00 | 3.07E-01 | -4.44E+00 | 9.06E-06 | 6.52E-05 |
| **ssc-miR-130b** | 2.64E+01 | -1.40E+00 | 3.69E-01 | -3.78E+00 | 1.59E-04 | 7.93E-04 |
| **ssc-miR-421-3p** | 1.32E+02 | -1.43E+00 | 2.30E-01 | -6.20E+00 | 5.82E-10 | 1.75E-08 |
| **ssc-let-7e** | 3.46E+03 | -1.50E+00 | 2.19E-01 | -6.84E+00 | 7.67E-12 | 3.45E-10 |
| **ssc-miR-7139-3p** | 6.55E+00 | -1.57E+00 | 6.55E-01 | -2.40E+00 | 1.65E-02 | 4.63E-02 |
| **ssc-miR-664-5p** | 2.66E+01 | -1.94E+00 | 3.63E-01 | -5.33E+00 | 9.58E-08 | 1.72E-06 |
| **ssc-miR-202-3p** | 5.75E+02 | -2.19E+00 | 2.32E-01 | -9.44E+00 | 3.83E-21 | 3.45E-19 |
| **ssc-miR-423-5p** | 3.74E+03 | -2.57E+00 | 3.43E-01 | -7.51E+00 | 6.03E-14 | 3.62E-12 |
| **ssc-miR-219a** | 8.09E+00 | -3.35E+00 | 7.59E-01 | -4.41E+00 | 1.04E-05 | 6.72E-05 |
| **ssc-miR-27b-5p** | 1.05E+02 | -3.40E+00 | 2.83E-01 | -1.20E+01 | 3.85E-33 | 6.94E-31 |

***Supplemental Table 3.*** *Genomic targets of nuclear miRNA.*

| Gene symbol | NCBI Gene ID | microRNA | Direction | Position | Strand | Length | MFE | p-value |
| --- | --- | --- | --- | --- | --- | --- | --- | --- |
| RASGRF2 | 5924 | ssc-miR-27b-5p | Sense | -374 | + | 21 | -30.8 | 0.03 |
| RBKS | 64080 | ssc-miR-27b-5p | Antisense | -463 | + | 22 | -31.0 | 0.03 |
| CA14 | 23632 | ssc-miR-27b-5p | Sense | -158 | + | 19 | -30.8 | 0.03 |
| DCX | 1641 | ssc-miR-27b-5p | Sense | -1460 | + | 26 | -31.8 | 0.02 |
| SP3 | 6670 | ssc-miR-423-5p | Antisense | -805 | + | 25 | -36.3 | 0.02 |
| JOSD2 | 126119 | ssc-miR-423-5p | Sense | -1100 | - | 22 | -35.1 | 0.04 |
| JOSD2 | 126119 | ssc-miR-423-5p | Sense | -1296 | - | 22 | -35.1 | 0.04 |
| NKX2-1 | 7080 | ssc-miR-423-5p | Sense | -36 | - | 22 | -39.9 | 0.01 |
| LDB1 | 8861 | ssc-miR-423-5p | Antisense | -8 | + | 23 | -35.1 | 0.04 |
| RUSC1 | 23623 | ssc-miR-423-5p | Sense | -424 | + | 24 | -34.4 | 0.05 |
| RUSC1 | 23623 | ssc-miR-423-5p | Sense | -914 | + | 24 | -34.4 | 0.05 |
| CCND1 | 595 | ssc-miR-423-5p | Sense | -108 | + | 24 | -34.7 | 0.04 |
| GAPDH | 2597 | ssc-miR-423-5p | Sense | -712 | + | 19 | -38.5 | 0.01 |
| MMP14 | 4323 | ssc-miR-423-5p | Antisense | -8 | - | 26 | -35.9 | 0.03 |
| KCNAB3 | 9196 | ssc-miR-423-5p | Sense | -1191 | - | 19 | -39.3 | 0.01 |
| AHDC1 | 27245 | ssc-miR-423-5p | Antisense | -198 | + | 22 | -35.2 | 0.03 |
| RTN4 | 57142 | ssc-let-7e-5p | Antisense | -1263 | + | 20 | -29.7 | 0.05 |
| SP3 | 6670 | ssc-let-7e-5p | Antisense | -808 | + | 19 | -31.2 | 0.03 |
| MSL1 | 339287 | ssc-let-7e-5p | Sense | -191 | + | 25 | -33.6 | 0.01 |
| IPO4 | 79711 | ssc-let-7e-5p | Sense | -1465 | - | 25 | -31.4 | 0.03 |
| UBTF | 7343 | ssc-let-7e-5p | Antisense | -675 | + | 17 | -30.2 | 0.04 |
| UBTF | 7343 | ssc-let-7e-5p | Antisense | -1884 | + | 17 | -30.2 | 0.04 |
| C3orf58 | 205428 | ssc-let-7e-5p | Sense | -408 | + | 25 | -32.3 | 0.02 |
| TFDP2 | 7029 | ssc-let-7e-5p | Sense | -598 | - | 18 | -30.6 | 0.04 |
| SMARCD3 | 6604 | ssc-miR-421 | Antisense | -153 | + | 25 | -26.4 | 0.04 |
| STMN1 | 3925 | ssc-miR-421 | Sense | -270 | - | 20 | -31.8 | 0.0 |
| SP3 | 6670 | ssc-let-7d-5p | Antisense | -808 | + | 20 | -30.3 | 0.04 |
| PPP2R2B | 5521 | ssc-let-7d-5p | Sense | -65 | - | 16 | -29.9 | 0.05 |
| PPP2R2B | 5521 | ssc-let-7d-5p | Sense | -254 | - | 16 | -29.9 | 0.05 |
| MSL1 | 339287 | ssc-let-7d-5p | Sense | -190 | + | 26 | -30.4 | 0.04 |
| CNOT6L | 246175 | ssc-miR-744 | Sense | -301 | - | 30 | -35.9 | 0.04 |
| NFKBIL1 | 4795 | ssc-miR-744 | Sense | -1327 | + | 27 | -39.4 | 0.01 |
| PORCN | 64840 | ssc-miR-744 | Sense | -764 | + | 23 | -36.2 | 0.04 |
| CIC | 23152 | ssc-miR-744 | Antisense | -420 | - | 26 | -38.5 | 0.02 |
| CIC | 23152 | ssc-miR-744 | Antisense | -1014 | - | 24 | -36.6 | 0.04 |
| FNDC5 | 252995 | ssc-miR-339-5p | Antisense | -132 | + | 25 | -37.8 | 0.02 |
| FMNL2 | 114793 | ssc-miR-339-5p | Sense | -115 | + | 30 | -37.7 | 0.01 |
| REPIN1 | 29803 | ssc-miR-339-5p | Sense | -1444 | + | 26 | -37.4 | 0.01 |
| PIK3CA | 5290 | ssc-miR-339-5p | Sense | -39 | + | 26 | -36.6 | 0.02 |
| SAP130 | 79595 | ssc-miR-339-5p | Sense | -21 | - | 27 | -35.1 | 0.03 |
| RNF208 | 727800 | ssc-miR-339-5p | Antisense | -291 | + | 28 | -37.3 | 0.02 |
| FNDC3B | 64778 | ssc-miR-339-5p | Antisense | -53 | - | 31 | -37.6 | 0.02 |
| SRA1 | 10011 | ssc-miR-339-5p | Sense | -142 | - | 28 | -34.3 | 0.03 |
| SRA1 | 10011 | ssc-miR-339-5p | Sense | -779 | - | 28 | -34.3 | 0.03 |
| SRA1 | 10011 | ssc-miR-339-5p | Antisense | -229 | + | 33 | -33.7 | 0.04 |
| SRA1 | 10011 | ssc-miR-339-5p | Antisense | -866 | + | 33 | -33.7 | 0.04 |
| MAZ | 4150 | ssc-miR-769-5p | Sense | -698 | + | 32 | -32.7 | 0.04 |
| ZNF496 | 84838 | ssc-miR-769-5p | Antisense | -167 | + | 24 | -33.3 | 0.04 |
| LMOD3 | 56203 | ssc-miR-19b-3p | Sense | -73 | - | 21 |  | 0.02 |
| ROGDI | 79641 | ssc-miR-19b-3p | Sense | -1464 | - | 26 | -25.5 | 0.03 |
| SULF1 | 23213 | ssc-miR-19b-3p | Sense | -143 | + | 26 | -24.4 | 0.05 |
| CHD3 | 1107 | ssc-miR-19b-3p | Antisense | -1295 | - | 24 | -24.8 | 0.05 |
| MEF2D | 4209 | ssc-miR-19b-3p | Antisense | -1182 | + | 25 | -28.9 | 0.01 |
| PREX2 | 80243 | ssc-miR-19b-3p | Sense | -589 | + | 23 | -24.4 | 0.05 |
| ZNF646 | 9726 | ssc-miR-19b-3p | Sense | -22 | + | 26 | -24.8 | 0.04 |
